# Impaired Toll-like receptor 2-mediated Th1 and Th17/22 cytokines secretion in human peripheral blood mononuclear cells from patients with atopic dermatitis

**DOI:** 10.1186/s12967-015-0744-1

**Published:** 2015-12-18

**Authors:** Yangyang Yu, Yarui Zhang, Jie Zhang, Xia Dou, Hong Yang, Yong Shao, Kepeng Wang, Bo Yu, Wei Zhang, Hang Yung Alaster Lau

**Affiliations:** School of Medicine, Shenzhen University, Shenzhen, Guangdong China; Shenzhen Key Laboratory for Translational Medicine of Dermatology, Biomedical Research Institute, Shenzhen Peking University-The Hong Kong University of Science and Technology Medical Center, No. 1120, Lianhua Road, Futian District, Shenzhen, 518036 Guangdong China; Department of Dermatology, Peking University Shenzhen Hospital, Shenzhen, Guangdong China; Department of Clinical Laboratory, Peking University Shenzhen Hospital, Shenzhen, Guangdong China; School of Biomedical Sciences, Faculty of Medicine, The Chinese University of Hong Kong, Hong Kong, SAR, China

**Keywords:** Atopic dermatitis, Toll-like receptor 2, Th1 cytokine, Th2 cytokine, Th17/22 cytokines

## Abstract

**Background:**

Impaired Toll-like receptor 2 (TLR2) function has been associated with the pathogenesis of atopic dermatitis (AD). However, there are only few studies reporting on the TLR2-induced immunological responses of circulating leucocytes of AD patients. We thus investigated the expression and secretion of Th1, Th2 and Th17/22 cytokines triggered by TLR2 ligands in human peripheral blood mononuclear cells (PBMCs) from AD patients. Expression of TLR2, 1, 6 and high-affinity receptor for IgE (FcεRI) were further investigated to evaluate the outcome of immune response in AD.

**Methods:**

Expression of TLR2, 1, 6 and FcεRI in PBMCs from AD patients and healthy individuals were measured by qPCR. Subsequent to stimulation with TLR2 ligands PGN and Pam3CSK4, expression and secretion of Th1, Th2 and Th17/22 cytokines were investigated by qPCR and ELISA.

**Results:**

The levels of TLR2, 1, 6 mRNA were not altered in both groups of subjects while that of FcεRI was increased in AD patients. Subsequent to the activation by TLR2 ligands, PBMCs from AD patients significantly released less IFN-γ, IL-17F and IL-22 than those from healthy controls while no detectable level of release was observed with the other cytokines. In contrast, significantly higher levels of mRNA expression for TNF-α, IL5, IL-17A and IL-22 were observed in TLR2 activated PBMCs of AD patients than those of healthy control.

**Conclusions:**

PBMCs from AD patients are defective in the secretion of Th1 and Th17/22 cytokines in response to TLR2 ligands. The inconsistent increased expression of the mRNA for the corresponding Th1 cytokines and the Th2 cytokines IL-5 suggested that there may be alterations of downstream signaling events in the cytokine release mechanisms of PBMCs that are associated with the development of AD.

## Background

Atopic dermatitis (AD) is the most common chronic inflammatory skin disease which affects 10–20 % children and 1–3 % adults worldwide [1]. The pathogenesis of AD is multifactorial with complex genetic and environmental influences. However, patients with AD have frequent bacterial skin infection indicating that immune abnormalities, especially innate immune dysfunctions, are critical for the pathogenesis of AD. Over 90 % of AD patients are colonized with Staphylococcus aureus (*S. aureus*) on lesional and non-lesional skin [2]. *S. aureus* could induce persistent inflammation in AD patients and anti-staphylococcal treatments are beneficial for the amelioration of disease severity [2, 3]. The Toll-like receptors (TLRs) family which belongs to the pattern-recognition molecules/receptors (PRRs) is at the center of detection mechanisms for the innate immunity. TLR2 recognizes *S. aureus* and is responsible for host defense against *S. aureus* infection [4]. In this regard, dysfunction in TLR2 signaling has been suggested to be involved in the development of AD.

Patients carrying a R753Q mutation in TLR2 or with TLR2–16934A > T polymorphism frequently develop severe AD [5, 6]. Furthermore, TLR2-mediated release of inflammatory mediators from immune effector cells (T cells, B cells, mast cells, eosinophils, macrophages and dendritic cells) and structural cells (keratinocytes and epithelial cells) are defective in AD patients, and thus partially explained the increased susceptibility to pathogens infection. These inflammatory mediators include TNF, IL-1, IL-6 and IL-8 [7–9]. However, the modulatory effect of TLR2 activation on priming the immune response in AD remains unclear.

It has been shown that the immunological milieu of AD is dominated by Th2-skewed immune response [10]. Augmentation of Th2 response could be the result of enhanced Th2 signaling or deficient Th1 response. However, there is no systematic analysis on the Th1- and Th2-derived cytokines in TLR2-mediated immune response in AD patients. Meanwhile, Th17/22 cytokines are recently indicated to regulate the pathogenesis of AD by regulating the expression of various cytokines and chemokines [3, 11]. However, whether Th17/22 response to TLR2 stimuli is impaired in AD patients is not yet known. In this study, we employed PBMCs isolated from healthy controls or AD patients to examine the expression and secretion profile of T helper cytokines in these cells upon TLR2 activation. Two classical TLR2 ligands peptidoglycan (PGN) and tripalmitoyl-S-glycero-Cys-(Lys)4 (Pam3CSK4) were applied in the investigation. While PGN is a primary cell wall component of Gram-positive bacteria which activates TLR2 and TLR6 heterodimer, Pam3CSK4 is a synthetic tripalmitoylated lipopeptide which promotes the formation of TLR2 and TLR1 heterodimer [4]. In general, we observed that PBMCs from AD patients showed reduced secretion of Th1 and Th17/22 cytokines, whereas the mRNA expression of the Th2 cytokine IL-5 and the AD-related receptor FcεRI were increased. Such change may cope with the pathogenesis of AD in humans in the context of distorted balance between immune cell mediated inflammation and infection clearance.

## Methods

### Blood samples

Heparinized blood was obtained from 40 patients suffering from AD according to the criteria of Hanifin and Rajka (27 female, 13 male, mean age 31 years, range 20–45 years) with moderate to severe disease activity [12], while 43 healthy individuals (29 female, 14 male, mean age 29 years, range 20–42 years) served as controls. Study procedures were reviewed and approved by ethics committee of Peking University Shenzhen Hospital Board.

### Culture and stimulation of PBMCs

PBMCs were separated from peripheral blood by Ficoll–Hypaque density gradient centrifugation. Cells were suspended at 1 × 10^6^/mL in complete culture medium (RPMI 1640 supplemented with 2 mmol/L l-glutamine, penicillin–streptomycin, 10 % fetal bovine serum (all from Invitrogen, Carlsbad, USA) and 55 μM 2-Mercaptoethanol (GIBCO, Eggestein, Germany). PBMCs were stimulated with 1 μg/mL PGN from *S. aureus* (Sigma-Aldrich, Deisenhofen, Germany) or Pam3CSK4 (Invivogen, San Diego, California, USA) for 8 h or 48 h for the RT-PCR and immunoassay studies respectively. These time points were determined by preliminary time course studies which demonstrated optimal mRNA expression and protein secretion of cytokines respectively.

### RNA extraction and qRT-PCR

At the end of the 8 h incubation with TLR2 ligands, cell pellets were collected by centrifugation and were then lysed with TRIzol reagent for the extraction of RNA. Total RNA was isolated with TRIzol reagent and was quantified by measuring the ratio of A_260nm_/A_280nm_. RNA (1 μg) was then reverse transcribed with the Revert Aid First Strand cDNA Synthesis Kit (Thermo scientific, Waltham, MA, USA). The RT mixture was incubated for 50 min at 42 °C followed by 15 min at 70 °C. qPCR was performed using the SYBR Green Dye method which was carried out using cDNAs supplemented with SYBR Green supermix (Bio-rad, Hercules, CA, USA) and 100 nM paired primers for different genes to be analyzed in PCR buffer. The PCR protocol consisted of a cycle at 95 °C for 5 min followed by 40 cycles consisting of 15 s at 95 °C, 30 s at 60 °C and 30 s at 72 °C. The 2^−△△Ct^ method was used for quantification of the target gene expression. All tests were done in triplicates. The average Ct was calculated for the target genes and internal control (GAPDH) and the ΔCt (Ct,_target_ − Ct,_GAPDH_) values were determined. Fold expression change of target genes in AD patients was determined relative to controls as 2^−ΔΔCt^, where ΔΔCt = ΔCt (patient or control) − ΔCt (average for controls).

### Immunoassays

Cell culture medium was collected after 48 h incubation. Secretion of TNF-α, IFN-γ, IL-4, IL-5, IL-12, IL-17A, IL-17F, IL-18, IL-22, IL-31, IL-33 and TSLP from PBMCs was determined by commercial enzyme-linked immunosorbent assay (ELISA) kits (eBioscience, San Diego, CA, USA and PeproTech, New Jersey, USA). Spontaneous release of the above cytokines was undetectable in both healthy controls and AD patients.

### Statistical analysis

Statistical significance was determined by using an unpaired student *t* test to compare samples from patients with those of healthy individuals. Differences were considered significant at a P value of less than 0.05.

## Results

### Altered Th1 cytokines production in PBMCs from AD patients upon stimulation with TLR2 ligands

**Fig. 1:**
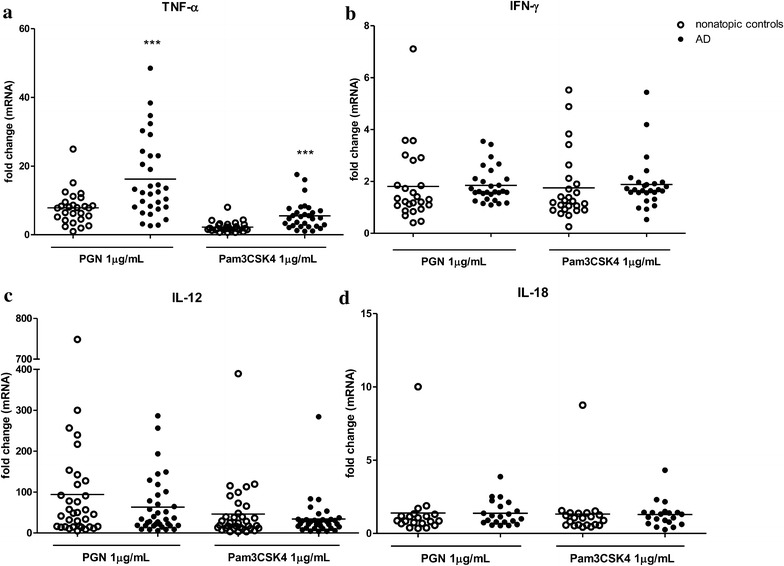
Effects of Toll-like receptor 2 (TLR2) ligands on mRNA expression of Th1 cytokines from human peripheral blood mononuclear cells (PBMCs) in atopic dermatitis (AD) patients. PBMCs were incubated with PGN or Pam3CSK4 for 8 h. TNF-α (**a**), IFN-γ (**b**), IL-12 (**c**) and IL-18 (**d**) mRNA expression from PBMCs was determined by qPCR. Data were shown as 2^−ΔΔCt^ ± SEM. T-test was employed to compare the significant difference between AD patients and healthy individuals. ***p < 0.001

To investigate the role of TLR2 activation in AD development and inflammation, we examined the expression and secretion of inflammatory cytokines by PBMCs upon TLR2 activation. After incubation with TLR2 ligands for 8 h, the level of TNF-α mRNA expression in PBMCs from AD patients was significantly higher than that from healthy individuals (Fig. 1a). In contrast, TLR2 ligands stimulation did not alter the mRNA expression of IFN-γ, IL-12 and IL-18 in PBMCs of AD patients when compared with those of healthy controls (Fig. 1b–d).

**Fig. 2:**
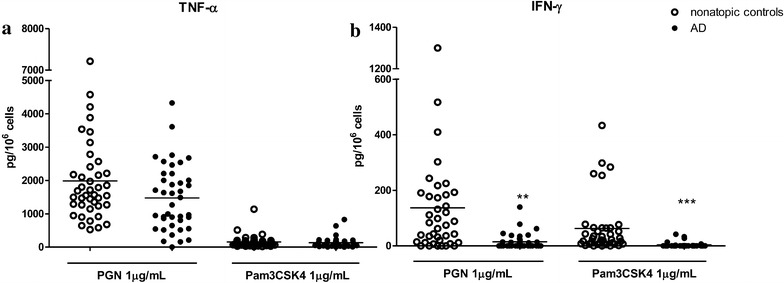
Effects of TLR2 ligands on the release of Th1 cytokines from PBMCs in AD patients. PBMCs were incubated with PGN or Pam3CSK4 for 48. Release of TNF-α (**a**) and IFN-γ (**b**) from PBMCs was determined by ELISA. Data are shown as mean ± SEM. T-test was employed to compare the significant difference between AD patients and healthy individuals. **p < 0.01, ***p < 0.001

Incubation of PBMC with TLR2 ligands for 48 h triggered the release of IFN-γ and TNF-α, but not IL-12 and IL-18. The levels of release of these two cytokines induced by PGN were higher than those induced by Pam3CSK4. Furthermore, the release of IFN-γ from PBMCs stimulated with both TLR2 ligands was almost abolished in AD patients, whereas no significant difference was observed in the release of TNF-α between AD patients and healthy individuals (Fig. 2).

### Augmented production of Th2 cytokine in PBMCs from AD patients upon stimulation with TLR2 ligands

**Fig. 3:**
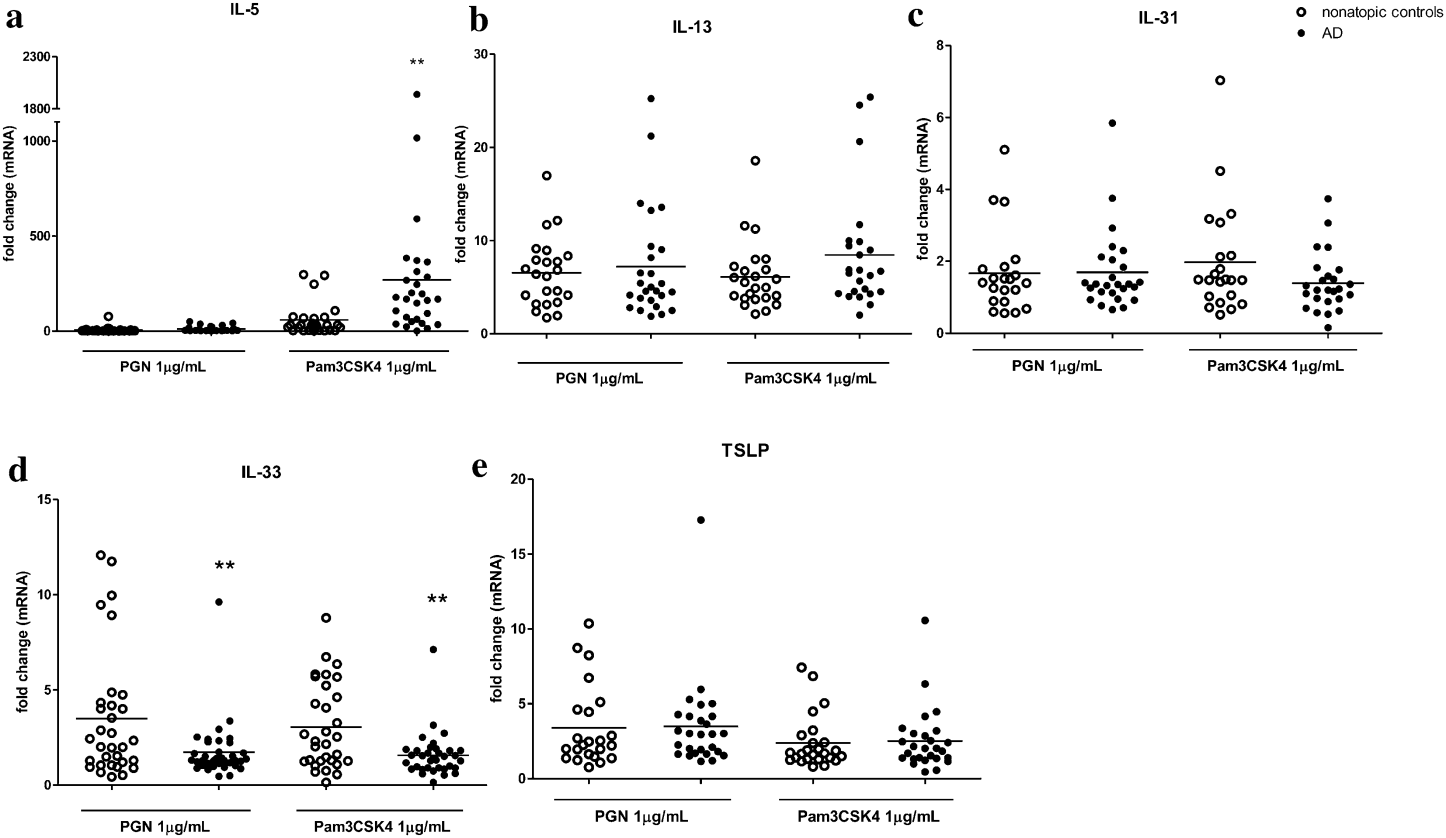
Effects of TLR2 ligands on mRNA expression of Th2 cytokines from PBMCs in AD patients. PBMCs were incubated with PGN or Pam3CSK4 for 8 h. IL-5 (**a**), IL-13 (**b**), IL-31 (**c**), IL-33 (**d**) and TSLP (**e**) mRNA expression from PBMCs was determined by qPCR. Data were shown as 2^−ΔΔCt^ ± SEM. T-test was employed to compare the significant difference between AD patients and healthy individuals. **p < 0.01

Among the Th2 cytokines studied, the mRNA level of IL-13, IL-31 and TSLP were comparable between the PMBCs of both AD patients and healthy control (Fig. 3b, c, e). In contrast, the level of IL-33 mRNA was less upregulated by TLR2 signaling in AD patients than that in healthy controls (Fig. 3d) while TLR2/1 ligand Pam3CSK4 induced dramatically higher level of IL-5 mRNA in AD patients (Fig. 3a). The basal level of IL-4 mRNA in PBMCs was undetectable in AD patients and healthy individuals (using four different primers specifically targeted for IL-4), nor did TLR2 activation triggered the expression of IL-4 (data not shown).

Neither of the TLR2 ligand triggered the release of IL-4, IL-5, IL-31, IL-33 or TSLP from PBMCs in AD patients and healthy controls (data not shown).

### Altered Th17/22 cytokines production in PBMCs from AD patients upon stimulation with TLR2 ligands

**Fig. 4:**
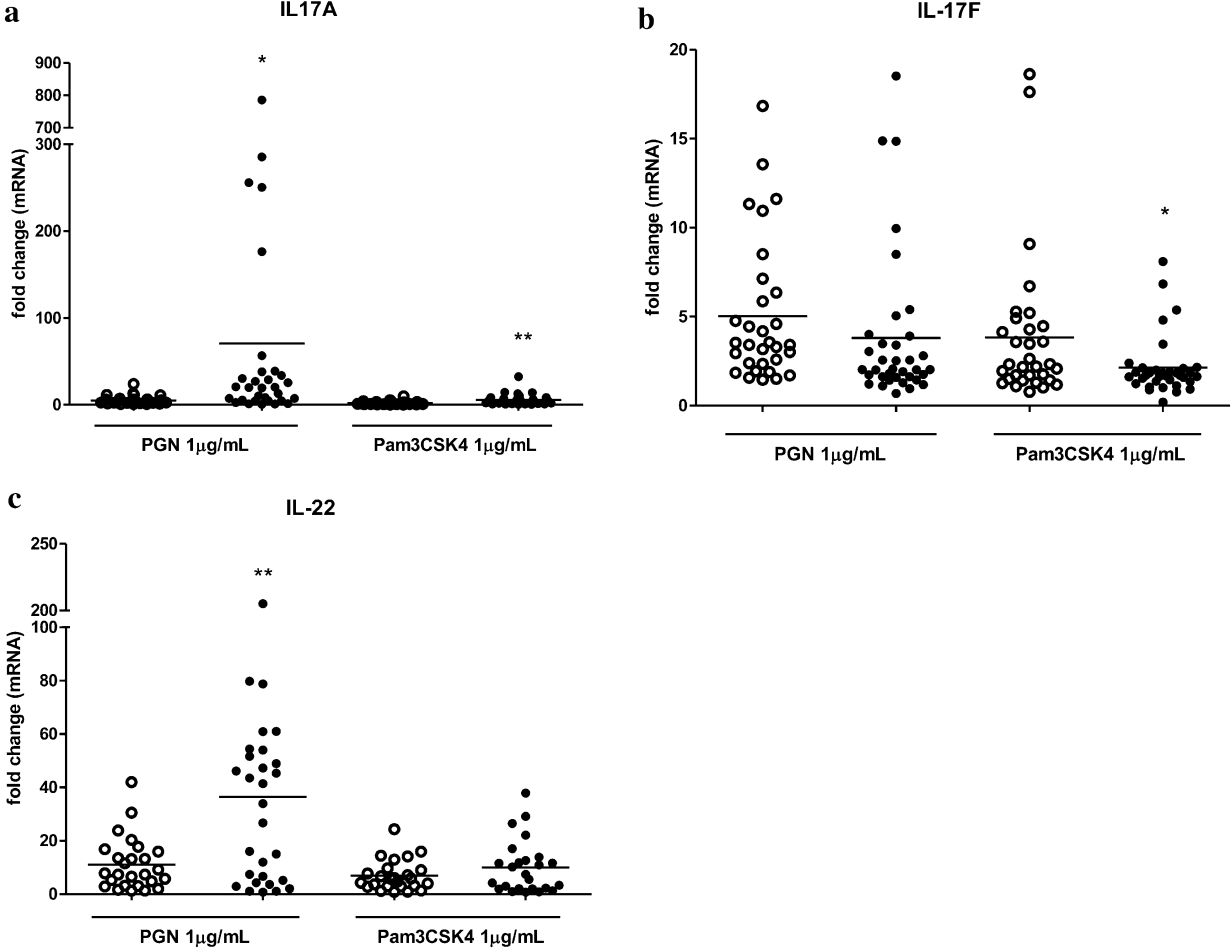
Effects of TLR2 ligands on mRNA expression of Th17/22 cytokines from PBMCs in AD patients. PBMCs were incubated with PGN or Pam3CSK4 for 8 h. IL-17A (**a**), IL-17F (**b**) and IL-22 (**c**) mRNA expression from PBMCs was determined by qPCR. Data were shown as 2^−ΔΔCt^ ± SEM. T-test was employed to compare the significant difference between AD patients and healthy individuals. *p < 0.05, **p < 0.01

PGN induced significantly higher mRNA expression of IL-17A and IL-22 in PBMCs of AD patients (Fig. 4a, c) while, both TLR2-ligands reduced the expression of IL-17F mRNA in PBMCs isolated from AD patients than that from healthy controls (Fig. 4b).

**Fig. 5:**
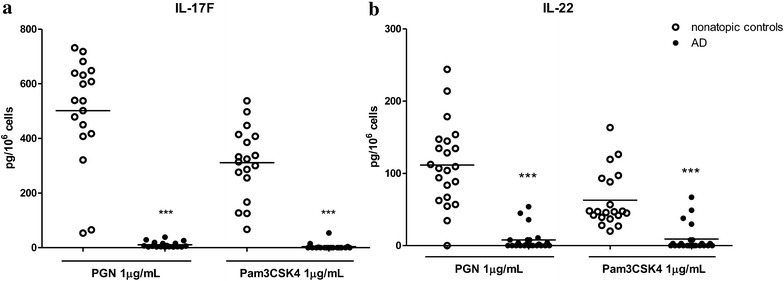
Effect of TLR2 ligands on the release of Th17/22 cytokines from PBMCs in AD patients. PBMCs were incubated with PGN or Pam3CSK4 for 48 h. Release of IL-17F (**a**) and IL-22 (**b**) from PBMCs was determined by ELISA. Data are shown as mean ± SEM. T-test was employed to compare the significant difference between AD patients and healthy individuals. ***p < 0.001

Secretion of IL-17F and IL-22 from AD patient derived PBMCs upon TLR2 activation was significantly lower. There was no release of IL-17A from PBMCs stimulated with TLR2 ligands in both groups (Fig. 5).

### No change of TLR2, TLR1 and TLR6 expression from PBMCs in AD patients

**Fig. 6:**
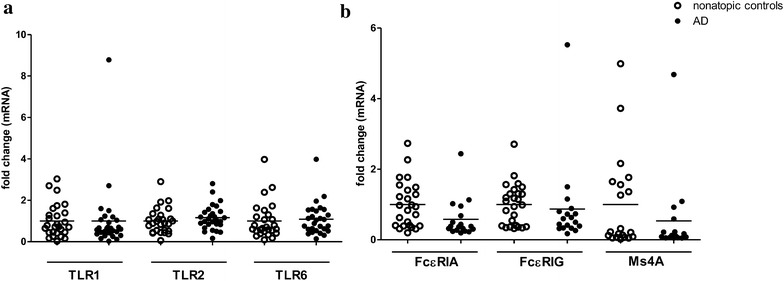
Quantitative analysis of mRNA expression of TLR2, 1, 6 (**a**) and subunits of FcεRI (**b**) from PBMCs in AD patients. Their relative expression was calculated using the 2^−ΔΔCt^ method. Data were shown as 2^−ΔΔCt^ ± SEM. T-test was employed to compare the significant difference between AD patients and healthy individuals

Reduced TLR2 expression was observed in many other cells types including macrophage, T cells, skin epithelium in AD patients [8, 9, 13]. However, the mRNA expression level of TLR2, TLR1 and TLR6 from PBMCs was not altered in AD patients compared with healthy controls (Fig. 6a).

### Enhanced expression of FcεRI from PBMCs upon stimulation with TLR2 ligands

**Fig. 7:**
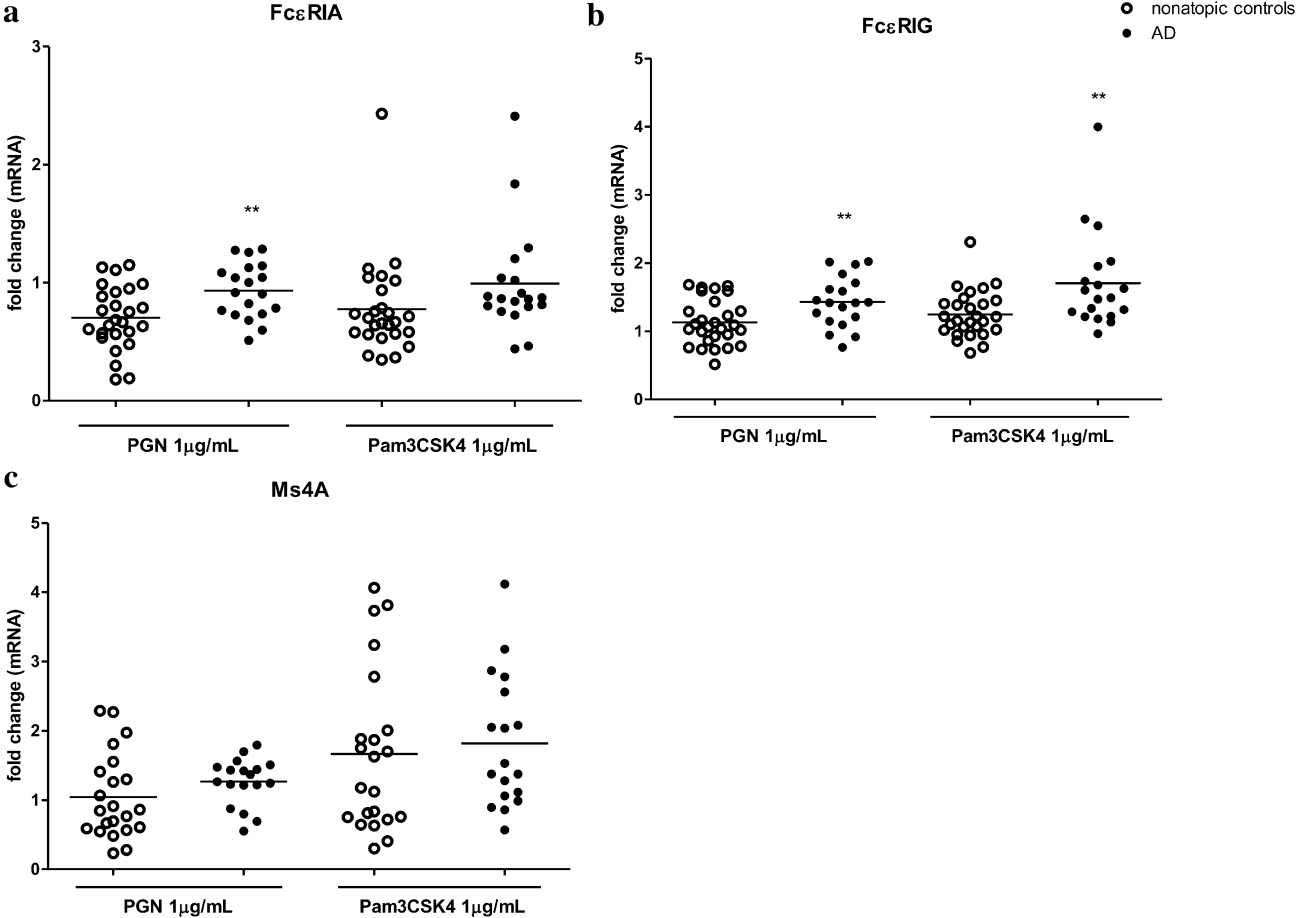
Effect of TLR2 ligands on mRNA expression of subunits of FcεRI from PBMCs in AD patients. PBMCs were incubated with PGN or Pam3CSK4 for 8 h. FcεRIA (**a**), FcεRIG (**b**) and Ms4A (**c**) mRNA expression from PBMCs was determined by qPCR. Data were shown as 2^−ΔΔCt^ ± SEM. T-test was employed to compare the significant difference between AD patients and healthy individuals. **p < 0.01

The high-affinity receptor for IgE (FcεRI) is key component in the induction of AD [14]. In AD patients, basal mRNA levels of the three FcεRI receptor subunits (FcεRIA, FcεRIG and Ms4A) were not altered in AD patients and healthy individuals (Fig. 6b). When stimulated with TLR2 ligands, mRNA levels of FcεRIA and FcεRIG in PBMCs were significantly higher in AD patients than those in healthy controls (Fig. 7a, b).

## Discussion

Increased susceptibility to skin *S. aureus* infection and defect in TLR2 signaling have been regarded as specific features reflecting the immune dysfunction in AD. A growing body of evidence suggests that innate immunity not only provides a rapid antimicrobial host defense, but also determines the type of subsequent acquired immune responses through directing the groups of cytokines released by activated immune cells [15]. In the current study, we observed a reduced Th1 and Th17/22 cytokines secretion, an enhanced mRNA expression of Th2 cytokine IL-5 and FcεRI from PBMCs upon TLR2 stimuli in AD patients. The different cellular responses demonstrated between AD patients and healthy controls suggested that initiation of innate immune reactions through TLR2 activation could eventually lead to a Th2-dominant inflammation in AD patients.

TLR2 is important in driving Th1 response to cutaneous sensitization through promoting the IFN-γ response to cutaneously introduced antigens [16]. However, we showed that TLR2-mediated IFN-γ secretion from PBMCs was almost abolished in AD patients. Consistent with our results, IFN-γ deficiency was generally observed in PBMCs at basal level or upon stimulation by other ligands including lipopolysaccharide (LPS), Staphylococcal enterotoxin B (SEB), house dust mite (HDM), Candida albicans (CA) and phytohaemagglutinin (PHA) in AD patients [17–19]. These observations confirm a general intrinsic cellular deficiency in IFN-γ response in PBMCs from patients with AD. IL-12 and IL-18 are inducers for IFN-γ expression [20], however, the mRNA expression IL-12 and IL-18 did not display noticeable difference from PBMCs triggered by TLR2 ligands in AD patients compared with healthy controls, nor did TLR2-ligands stimulated the release of the two cytokines in both groups. In contrast, a significantly reduced TLR2-mediated expression of IL-33 mRNA in PBMCs was observed in AD patients. IL-33 is a newly characterized cytokine belonging to the IL-1 cytokine family that drives Th2 response [21]. However, Seltmann’s group recently pointed out an important regulatory loop of IFN-γ and IL-33 in eczematous skin inflammation [22]. IFN-γ induced epithelial IL-33 expression which in turn acted on activated T cells to further improve the release of IFN-γ, indicating that IL-33 may act as a dual function cytokine which directs either Th1- or Th2-linked inflammation [22]. The impairment in TLR2-mediated IL-33 and IFN-γ production provides a potential mechanism for the deficiency in IFN-γ response in AD patients upon TLR2 activation.

Similar to the defect in IFN-γ production, impaired TLR2-mediated release of TNF-α from PBMCs was also observed in AD patients by other groups [7]. However, we showed that the level of TNF-α secretion from TLR2 ligands-stimulated PBMCs was slightly lower in AD patients, while the mRNA level of TNF-α elevated dramatically, implying that TLR2 signaling could be amplified in AD patients at the transcriptional level. Moreover, although the mRNA expression of IFN-γ from TLR2-ligands stimulated PBMCs was similar between AD patients and health control, the release of IFN-γ from PBMCs upon TLR2 activation was almost abolished in AD patients. The inconsistency in the mRNA expression and secretion of TNF-α and IFN-γ suggests a post-transcriptional or secretion defect in Th1 response in PBMCs from patients with AD.

Although TLR2 ligands did not induce the release of Th2 cytokines, it dramatically increased the IL-5 mRNA expression in AD patients. IL-5 is known to play a pivotal role as a major maturation and differentiation factor for eosinophils, which participate in AD pathogenesis [23]. Elevation in IL-5 mRNA expression would promote the Th2-skewed response in the context of the co-existence of other allergens such as house dust mite extracts, which could induce the release of IL-5 from PBMCs [19]. Although recent study has shown that TLR2 stimulation down-regulated FcεRI expression on epidermal Langerhans cells (LC) which play critical role in AD, we showed that TLR2-mediated FcεRI expression was elevated in PBMCs from AD patients. Allergen binding is mediated via IgE-bound FcεRI, which is highly expressed on epidermal dendritic cells, eosinophils, basophils and mast cells in AD, and once activated, triggers the release of Th2 cytokines including IL-5 [14, 24]. Synergistic effect of TLR2 activation on FcεRI-mediated response has been demonstrated in the literature [25, 26]. Furthermore, synergistic effects on FcεRI- and TLR-triggering could promote Th2 differentiation of human naive CD4^+^ cells [27]. In this regard, the augmented TLR2-mediated IL-5 and FcεRI mRNA expression in AD patients would further aggravate Th2-biased response in AD development in the co-existence of allergens which activate the FcεRI signaling.

Th17 cells produce Th17 and Th22 cytokines which are involved in the development of some autoimmune diseases [28]. Although Th17 cells are known to contribute to the pathogenesis of psoriasis, their roles in AD remain controversial. Increased number of Th17 cells was observed in the peripheral blood and skin lesion of patients with acute AD, whereas reduced IL-17 production was found in chronic AD patients [28, 29]. Furthermore, the decrease of circulating IFN-γ negative Th17 cells correlated with increased of CCL17, IgE and eosinophil levels in AD patients, indicating that decrease in circulating Th17 cells might contribute to the induction of AD [11]. The involvement of IL-17 immunity in the development of atopic march induced by enterotoxin B (SEB) from *S. aureus* has been first addressed by Zheng’group [30]. Epicutaneous exposure of SEB exaggerated systemic Th17/IL-17A immunity and enhanced allergic lung inflammation [30]. In the current study, TLR2-mediated release of IL-17F was abolished in AD patients while IL-17A release was not detected in either AD patients or healthy controls. However, TLR2 ligands trigger significantly larger amount of IL-17A mRNA expression in AD patients. Although IL-17A and IL-17F both belong to the IL-17 cytokine family and share the strongest sequence homology, recent studies have pointed out that IL-17A and IL-17F induce the release of different pro-inflammatory cytokines. In a mouse model of asthma, IL-17F^−/−^ mice exhibited enhanced Th2 response and eosinophil infiltration, while IL-17A^−/−^ mice showed the opposite, suggesting a suppressive role for IL-17F and a promotional role for IL-17A in the induction of asthma through regulating the Th2 response [31]. The impaired IL-17F response but enhanced IL-17A mRNA expression from PBMCs triggered by TLR2 ligands in AD patients may therefore contribute to a more complicated mechanism to the pathogenesis of AD through exacerbating the Th2 response in AD development.

On the other hand, the number of IL-22^+^CD8^+^T-cell is correlated with chronic AD disease severity [32]. However, we showed that TLR2-mediated IL-22 secretion was also severely impaired in AD patients. In contrast to the secretion of IL-22, the mRNA expression level of IL-22 was significantly higher in AD patients than that in healthy group, implying a post-transcriptional or secretion defect in PBMCs from AD patients. Such inconsistency of TLR2-mediated cytokine secretion and mRNA expression was observed in all the other cytokines examined including TNF-α, IFN-γ, and IL-17F where impairment in the cytokines secretion but enhancement or no change in mRNA expression were observed in AD patients compared with healthy controls. Consistent with our finding, impaired secretion but not mRNA expression of IL-6 and IL-8 was detected in macrophages isolated from PBMCs upon TLR2 stimulation in AD patients [9]. Taken together, we suggest that impairment of TLR2-mediated Th1 and Th17/22 cytokines secretion was more likely due to the defects in PBMC themselves in addition to the impairment in TLR2 signaling. Indeed, the phenotypical and functional defect of immune effector cells in AD patients have also been addressed by other groups [15, 17]. Natural killer (NK) cells and γδ^+^T cell were prone to undergo apoptosis on cell-to-cell contact with activated monocytes from patients with AD [15]. Similarly, reductions in the number of IFN-γ-producing cells and the amount of IFN-γ production per cell were observed in children with AD [17]. In this study, further investigation of TLR2, 1 and 6 mRNA expressions did not show any significant difference between AD patients and healthy controls suggesting that the impaired TLR2-mediated cytokines secretion in AD patients could not be explained by changes in TLR2 expression. On the other hand, studies have shown that defects in certain second messengers such as the cyclic AMP, protein kinase C and inositol involved in signaling systems have been suggested to provide a biochemical mechanism for the impairment of cytokines secretion seen in AD [33, 34]. Further studies are necessary to reveal the defects or dysfunctions of PBMCs at both the cellular and molecular levels in AD.

## Conclusions

In summary, TLR2-mediated responses were compromised in their capacity to secrete Th1 and Th17/22 cytokines. However, TLR2 activation enhanced the expression of Th2 cytokine IL-5 and FcεRI, thereby directing subsequent acquired immune responses toward a Th2-biased pattern, which in turn increases the risk for bacterial colonization and thus worsen clinical outcomes of patients as previously reported [35]. Furthermore, we showed for the first time the inconsistency in TLR2-mediated secretion and mRNA expression of Th1 and Th17/22 in PBMCs from AD patients, implying an intrinsic cellular defect or dysfunction of PBMCs themselves in the secretion of Th1 and Th17/22 cytokines in AD patients. Further investigation is required to elucidate the functional defect of PBMC from patients with AD to explain the imbalance in innate immunity as well as acquired immunity in AD development.
